# Comparative evaluation of CAR-expressing T-, NK-, NKT-cells, and macrophages in an immunocompetent mouse glioma model

**DOI:** 10.1093/noajnl/vdaf074

**Published:** 2025-04-12

**Authors:** Ryusuke Hatae, Payal B Watchmaker, Akane Yamamichi, Keith Kyewalabye, Kaori Okada, Su Phyu, Yitzhar Goretsky, Jeffrey Haegelin, Psalm Pineo-Cavanaugh, Marco Gallus, Lan Phung, Tiffany Chen, Haoyu Long, Pavlina Chuntova, David H Raulet, Masaki Terabe, Hideho Okada

**Affiliations:** Department of Neurological Surgery, University of California, San Francisco, San Francisco, California, USA; Department of Neurological Surgery, University of California, San Francisco, San Francisco, California, USA; Department of Neurological Surgery, University of California, San Francisco, San Francisco, California, USA; Department of Neurological Surgery, University of California, San Francisco, San Francisco, California, USA; Department of Neurological Surgery, University of California, San Francisco, San Francisco, California, USA; Department of Neurological Surgery, University of California, San Francisco, San Francisco, California, USA; Department of Neurological Surgery, University of California, San Francisco, San Francisco, California, USA; Department of Neurological Surgery, University of California, San Francisco, San Francisco, California, USA; Department of Neurological Surgery, University of California, San Francisco, San Francisco, California, USA; Department of Neurological Surgery, University of California, San Francisco, San Francisco, California, USA; Department of Neurological Surgery, University of California, San Francisco, San Francisco, California, USA; Department of Neurological Surgery, University of California, San Francisco, San Francisco, California, USA; Department of Neurological Surgery, University of California, San Francisco, San Francisco, California, USA; Department of Neurological Surgery, University of California, San Francisco, San Francisco, California, USA; Division of Immunology and Molecular Medicine, Department of Molecular and Cell Biology, University of California, Berkeley, Berkeley, California, USA; Neuro-Oncology Branch, National Cancer Institute, NIH, Bethesda, Maryland, USA; The Parker Institute for Cancer Immunotherapy, San Francisco, California, USA; Department of Neurological Surgery, University of California, San Francisco, San Francisco, California, USA

**Keywords:** CAR-NK cells, CAR-NKT cells, CAR-macrophage, combination CAR therapy, glioma syngeneic mouse models

## Abstract

**Background:**

While chimeric antigen receptor (CAR) T-cells are promising, there is a rapidly growing interest in developing other CAR-expressing immune cells. However, to date, no reported studies evaluated these cells side-by-side in immune-competent glioma models.

**Methods:**

We developed a novel C57BL/6-background transgenic mouse strain with all hematopoietic cells carrying the anti-epidermal growth factor receptor (EGFR)vIII-CAR downstream of a Lox-Stop-Lox cassette in the Rosa26 locus. Crossing with mice transgenic for Vav-Cre allowed the expression of anti-EGFRvIII CAR in all hematopoietic cells. In particular, we evaluated CAR-T, CAR-NKT, CAR-NK-cells, and CAR-macrophages in a syngeneic mouse SB28EGFRVIII glioma model.

**Results:**

CAR-NK and CAR-NKT-cells demonstrated anti-tumor effects comparable to CAR-T cells in vitro. A single intratumoral administration of CAR-T and CAR-NKT cells in combination mediated superior therapeutic efficacy compared to CAR-T cells or CAR-NKT-cells alone. A single intravenous infusion of CAR-NK cells following lymphodepletion failed to mediate significant anti-glioma effects. Additionally, intratumoral injection of CAR-NK cells did not confer therapeutic benefit. Contrary to previous reports using human macrophages, CAR-macrophages did not demonstrate enhanced antigen-presentation activity against glioma cells compared to non-CAR macrophages. Intratumorally administered CAR-macrophages failed to demonstrate local persistence or anti-tumor effects in vivo.

**Conclusions:**

These data provide a valuable basis as to which immune cells can mediate effective anti-glioma response in an immuno-competent glioma environment. Our data also suggest that a combination of CAR-T and CAR-NKT-cells may represent a promising therapeutic strategy.

**Key Points**

We developed a novel mouse model with CAR expression in all immune cells.

CAR-NKT combined with CAR-T produced high levels of IFN-γ and showed strong anti-tumor potential.

CAR-NK and CAR-macrophages failed to show in vivo efficacy.

**Importance of the Study**

This is the first study evaluating the efficacy of multiple CAR-expressing immune cell populations, including CAR-T, CAR-NKT, CAR-NK, and CAR-macrophages, in a syngeneic SB28 mouse glioma model. Most notably, our study demonstrates a superior therapeutic efficacy of CAR-T and CAR-NKT cells in combination compared to either cell type alone, suggesting that combination CAR therapy could be a promising treatment strategy. In contrast, CAR-NK cells displayed limited in vivo anti-tumor activity, and CAR-macrophages failed to show significant antigen-presentation or therapeutic benefits. As our SB28 syngeneic glioma model has clinically relevant immunological characteristics, these findings provide valuable insights into optimizing CAR-based immunotherapies for patients with glioma.

Chimeric antigen receptor T-cell (CAR-T) therapy has been shown to be effective in treating blood cancers. While recent phase I clinical studies reported promising signals at least transiently with local administration and multiple doses of CAR-T therapy for high-grade gliomas^1,2^ and diffuse midline gliomas,^3^ there are multiple challenges that need to be overcome to make CAR-T therapy effective for patients with malignant brain tumors.^4–6^

There also is a rapidly growing interest in evaluating alternative CAR-expressing immune cells, such as CAR-NKT cells, CAR-NK cells, and CAR-macrophages, each harboring unique attributes and potential advantages. Notably, CAR-NK and CAR-NKT-cells are being evaluated for “off-the-shelf” applications as allogeneic cell products, addressing a critical need for readily available therapeutic options.^7,8^ Also, the high abundance of naturally infiltrating myeloid cells in gliomas prompts a hypothesis that CAR-macrophages may mediate effective tropism and anti-glioma response. Early-phase clinical trials using CAR-NK-cells,^9^ CAR-NKT-cells,^10^ and CAR-macrophages^11^ are underway. However, pre-clinical data providing the basis for clinical trials have been mostly generated with immunocompromised mice bearing patient-derived xenografts (PDX) and receiving allogeneic human CAR cell products. To our knowledge, no prior pre-clinical studies have compared multiple CAR-expressing immune cell populations in syngeneic mouse models.

We previously developed a transgenic C57BL/6J mouse model in which all T-cells express a CAR-targeting the EGFRvIII mutation.^12^ This model enables the large-scale preparation of CAR-T cells with uniform quality, facilitating reproducible conditions and enhancing the reliability of pre-clinical data. Building upon this foundational work, we have now engineered a C57BL/6J mouse model that enables CAR expression in all immune cells, thereby allowing for the generation of CAR-T, CAR-NK, CAR-NKT, and CAR-myeloid cells among others. Employing this innovative model, we comprehensively evaluated the function of these CAR-expressing cells in syngeneic mice bearing EGFRvIII-positive intracerebral glioma.

In addition, we evaluated combination therapies using multiple CAR-expressing immune cell populations, which are readily available from our transgenic mice, to overcome the limited efficacy of CAR-T cells against solid tumors. This strategic approach marks a significant advancement in addressing the challenges in treating solid tumors, an area, where the solitary application of CAR-T-cell therapy has yet to fully demonstrate its therapeutic potential.

## Materials and Methods

### Mice and Cells

C57BL/6N mice were purchased from Jackson Laboratory (JAX 000664). Mice were approximately 9–10 weeks old during the experiment and maintained under specific pathogen-free conditions at the Animal Facility at UCSF, per an Institutional Animal Care and Use Committee-approved protocol.

The murine SB28 glioma and SB28 cells expressing murine EGFRvIII cell line were established in our labortaory.^13,14^ Cell lines were cultured in RPMI medium (Gibco) with 10% (v/v) heat-inactivated fetal bovine serum and 1% (v/v) penicillin-streptomycin mixed solution (Gibco, 15070063). Cell lines were free of mycoplasma contamination.

### The SB28-dual Minigene Cell Line

The SB28 murine glioma cell line was retrovirally transduced with a minigene cassette, including two peptides presented via the MHC I (gp100) and MHC-II (TRP-1) and expanded under blasticidin selection. After single-cell cloning, selected cells were cultured in cRPMI media containing 30 µg/µL blasticidin. The transduction of minigene was assayed and confirmed via PCR.

### In Vitro T-cell Cultures

The spleen was harvested to isolate CD3^+^ CAR-T cells from 8- to 12-week-old CAR-Transgenic mice. The spleen was minced and treated with ACK (Ammonium-Chloride-Potassium) Lysing buffer for 2 min to lyse the erythrocytes. CD3^+^ CAR-T cells were then purified from lymphocytes using MojoSort™ Mouse CD3 T cell Isolation Kit according to the manufacturer’s instructions (BioLegend, 480031). CAR-T cells were then activated for 2 days at 1 × 10^6^ cells per 24-well flat-bottomed plates with an equivalent number of CD3/CD28 washed Dynabeads (Gibco, 11453D), 30 U/mL hIL-2 (NIH) and 50 ng/mL mIL-15 (Peprotech, 21015) in 1 mL of complete RPMI [cRPMI: RPMI 1640 media with 10% FBS, 1% Penicillin-Streptomycin (Gibco, 15070063), 1% HEPES (Gibco, 15630080), 1% Glutamax (Gibco, 35050061), 1% non-essential amino acids (Gibco, 11140076), 1% sodium pyruvate (Gibco, 11360070), 0.5 mM 2-Mercaptoethanol (Gibco, 21985023)]. After stimulation, CAR-T was cultured for 7–10 days in a medium containing IL-2 and IL-15 (30 U/mL hIL-2, 50 ng/mL mIL-15) as an expansion step. Cell density was monitored daily and maintained in fresh media and cytokines at 0.5–1 × 10^6^ cells/mL.

### In Vitro Macrophage Cultures

For in vitro macrophage cultures, bone marrow cells were isolated from the femurs and tibiae of 8- to 12-week-old mice and incubated overnight in a treated plate, followed by stimulation with GM-CSF (20 ng/mL, Peprotech) in non-treated plates for 24 h before transduction. Concurrently, wells in a 6-well non-tissue culture-treated plate were coated with 20 µg/mL RetroNectin (Takara Bio USA, T100B) and stored at 4 °C overnight. For the transduction process, myeloid cells were resuspended in the virus to attain a concentration of 1 × 10^6/mL in 100% virus, with polybrene supplemented at 8 µg/mL. The myeloid cells, virus, and GM-CSF (20 ng/mL) were added to the RetroNectin-coated plates. The plates were centrifuged at 2200g at 37 °C for 45 min, and the cells were incubated overnight at 37 °C. The following morning, the virus-containing medium was gently removed, and fresh media and GM-CSF were added. Cell density was meticulously monitored daily and sustained in fresh media and cytokines at a concentration of 0.5–1 × 10^6 cells/mL.

### Ex Vivo Expansion of CAR-NK and WT NK-cells

Spleens were isolated from vav-cre CAR-Transgenic mice or control C57BL/6N CD45.1 under sterile conditions. Spleens were mashed, and a single-cell suspension was prepared to isolate NK-cells. NK cell Negative Isolation kit (Stemcell Technologies, Cat no: 19855) was used to enrich NK-cells. The enriched NK-cells were plated at 1e6 cells/mL in complete RPMI [cRPMI: RPMI 1640 media with 10% FBS, 1% Penicillin-Streptomycin (Gibco, 15070063), 1% HEPES (Gibco, 15630080), 1% Glutamax (Gibco, 35050061), 1% non-essential amino acids (Gibco, 11140076), 1% sodium pyruvate (Gibco, 11360070), 0.5 mM 2-Mercaptoethanol (Gibco, 21985023) with mIL12 (10 ng/mL, Peprotech) and mIL18 (50 ng/mL, Peprotech) for 16 h. After 16 h, NK-cells were washed and expanded further for 6 days in cRPMI media containing IL15 (100 ng/mL). On day 7, NK-cells (**Supplementary Figure 1B**) were used for in vivo or in vitro assays.

### In Vitro NKT-cell Cultures

For in vitro NKT-cell cultures, CAR-NKT-cells were isolated from the livers of 8- to 12-week-old CAR-Transgenic mice. The liver extraction involved an abdominal incision and placing a tube into the portal vein. The liver was infused slowly with 5 mL of Gibco liver perfusion buffer (cat#17701-038) at a rate of 1 mL/min. After infusion, the livers were harvested, washed with the same buffer to eliminate residual blood, and mechanically dissociated using a gentleMACS Dissociator, set to Program Spleen 1.01, for 1 min. This dissociation step was repeated for thorough tissue homogenization. The cells were then combined with a Gibco digestion buffer (cat#17703-34) and incubated at 37 °C in a water bath for 15 min with shaking for proper digestion. After filtering and washing, a Percoll gradient was employed to segregate the lymphocytes. The cells were stained with PE-labeled tetramer and anti-PE MicroBeads (Miltenyi Biotec, Cat #130-048-801). The PE-labeled mouse CD1d tetramers loaded with the α-GalCer analog PBS-57, were kindly provided by the NIH Tetramer Core Facility.

The isolated NKT-cells were then subjected to activation over 2 days in 96-well flat-bottomed plates, each well containing 2–5 × 10^5^ cells. This activation process utilized plate-bound CD3 antibody (17A2 Biolegend at 0.3 µg/mL), 0.3 µg/mL of CD28 antibody (37.51 Biolegend), and 50 U/mL of human IL-2 (from NIH), all in 0.2 mL of complete RPMI medium. Following this initial stimulation phase, the NKT-cells were cultured for 5–6 days in a medium supplemented with IL-2 (50 U/mL) to facilitate cell expansion. The cell density was monitored daily, ensuring optimal growth conditions by regularly refreshing the media and cytokines to maintain a concentration of 0.2–0.5 × 10^5^ cells per 0.2 mL. During a 3-week culture period, NKT-cells were periodically stimulated every week using CD3 and CD28 antibodies.

### In Vitro Co-culture

Macrophages (0.5–1 × 10^5^) were co-cultured with SB28 mEGFRvIII tumor cells at an effector: target ratio of 1:1 in 96-well flat-bottom non-treated plates in triplicate for two days in cRPMI. After 2 days, cells were collected, washed twice with PBS, and used in FACS analyses. The supernatant was also collected and used in ELISA analysis.

### Mouse Therapy Model

An aliquot of 1 × 10^4^ SB28 mEGFRvIII cells/mouse was stereotactically injected into the right hemisphere of anesthetized C57BL/6N mice (day 0). Tumor progression was evaluated by luminescence emission on Xenogen IVIS Spectrum after intraperitoneal injection of 1.5 mg of d-luciferin (GoldBio). Before treatment, mice were randomized, so the initial tumor burden in each group was equivalent. A combination of cyclophosphamide (3–4 mg/mouse) and fludarabine (1 mg/mouse) was intraperitoneally injected as lymphodepletion (LD) on day 10. Busulfan (25 mg/kg, Sigma-Aldrich) was intraperitoneally injected as myeloablative conditioning (MAC) for 5 days from day 7. Mice received intravenous (IV), intratumoral (IT), or intracerebroventricular (ICV) administration with immune cells for survival study the day after LD or MAC. For IT, we administered 2–3 µL of cells intrathecally through the same burr hole used for the intracranial tumor injection. For ICV, we administrated 5 µL of cells via a small burr hole (0.3 mm posterior to bregma; 1.0 mm lateral to the sagittal suture, depth 3 mm from skull).

### Cytotoxicity Assays

The cytotoxic effects of CAR-immune cells were evaluated using an xCELLigence system (Roche-Applied Science, Indianapolis, IN, USA). The immune cells were plated in at least triplicate on tumor cells at effector-to-target ratios (E:T) of 4:1. The group with tumor cells only served as the negative control, while tumor cells fully lysed by 0.1% Triton X-100 served as the positive control.

The culture plates were then inserted into the xCELLigence system and maintained at a constant temperature of 37 °C with a 5% carbon dioxide atmosphere. The system was set to monitor cell proliferation at 15-min intervals over a period ranging from 48 to 72 h.

## Results

### Establishment and Characterization of the C57BL/6-Background Vav-Cre CAR-Transgenic Mouse Model

**Figure 1.**
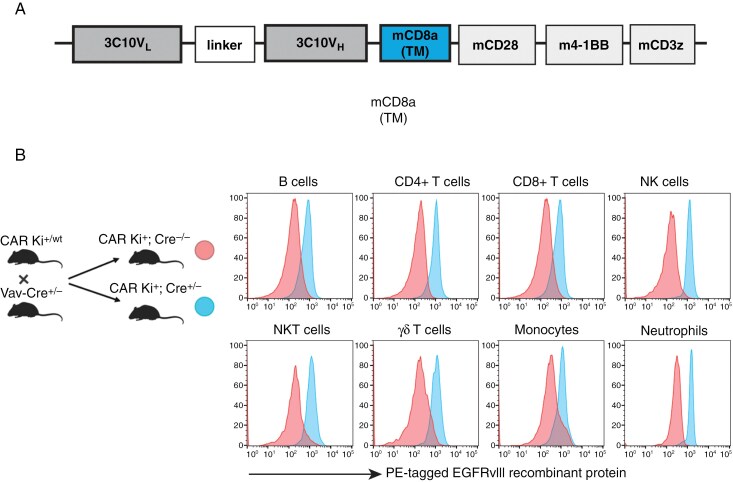
Diagram of the CAR construct and characterization of CAR expression in immune cell populations. (A) The CAR construct used in this study. (B) Expression of EGFRvIII-targeting CAR on different immune cell populations as measured by flow cytometry using conjugated recombinant EGFRvIIII protein.

We previously reported the development of the C57BL/6-background CD45.1^+^ CAR transgenic mouse strain carrying EGFRvIII-targeting CAR-T-cells.^12^ Briefly, we engineered C57BL/6J background mice to incorporate the anti-EGFRvIII-CAR construct (**Figure 1A**), positioned downstream of a Lox-Stop-Lox cassette within the Rosa26 locus (CAR Ki+/wt). Subsequently, we crossbred these mice with CD4-Cre transgenic mice, enabling the specific expression of the CAR construct in T-cells.^12^

In the current study, we crossbred our CAR Ki+/wt mice with Vav-Cre transgenic mice, which drove Cre expression across all hematopoietic cells,^15–17^ resulting in the generation of CAR Ki+; Cre+/- mice, wherein CAR was expressed on T-cells, B-cells, NK-cells, NKT-cells, monocytes, and neutrophils (Figure 1B**and****Supplementary Figure 1A****for gating schema**). We used this versatile model to study the role of each CAR-expressing cell population, and we described the differentiation and culture methods in **Supplementary Figure 2A and B**.

### Despite Their Effective In Vitro Cytotoxicity, Memory-like CAR-NK Cells Fail to Suppress Glioma Growth In Vivo

To generate potent CAR-NK cells, we employed a method to induce memory-like NK cells by pre-activating splenic NK cells overnight with a combination of cytokines, IL-12 and IL-18, and subsequently expanded with IL-15 for 5 days^18^ (**Supplementary Figure 2A**). Previous studies have demonstrated effective induction of memory-like NK-cells following viral infections,^19^ hapten-induced contact hypersensitivity,^20^ and exposure to inflammatory cytokines in the absence of defined antigens.^18^ Cytokine-induced memory NK-cells mediated effective anti-tumor effects in vivo in pre-clinical and clinical studies.^21,22^ However, to date, all the published pre-clinical murine studies with CAR–NK cells have been performed in immunodeficient mice with human NK-cells and relied on the use of IL2 or IL15.^23^ To our knowledge, ours is the first study that assessed the therapeutic efficacy of mouse CAR-NK cells in a solid tumor setting using immunocompetent mice.

**Figure 2.**
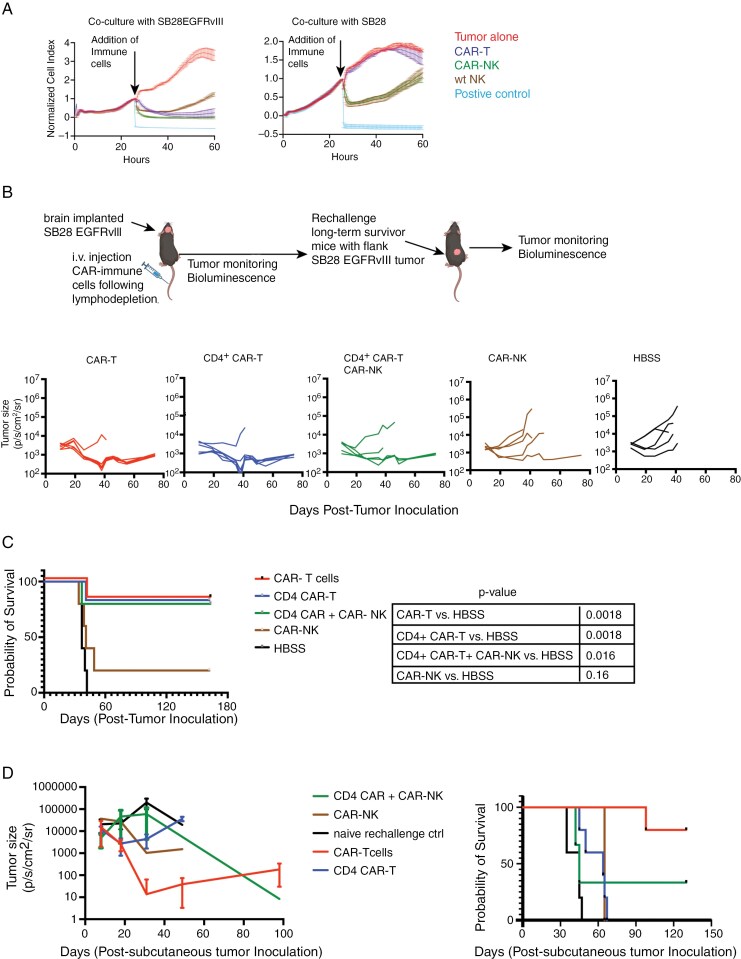
Cytolytic activity of anti-EGFRvIII CAR-NK compared to CAR-T cells in vitro and in vivo. (A) In vitro, the cytolytic function of CAR-NK-cells was assessed using xCELLigence assay. Ratio of target to effector cells was 1:4. Unpaired t-test between wt NK and CAR-NK against SB28EGFRvIII glioma cells, *P* < .0001, at time-point 58 h. (B) C57BL6/J mice bearing intracerebral SB28EGFRvIII glioma underwent lymphodepletion on Day 10 and received a single IV infusion of 3 × 10^6^ CAR-T (*n* = 6), CD4 CAR-T (*n* = 6), CAR-NK cells (*n* = 5) or a combination of CD4^+^ CAR (3 × 10^6^ cells) and CAR-NK (3 × 10^6^ cells) (*n* = 5) cells on Day 11. Longitudinal bioluminescence imaging (BLI) was performed to monitor tumor size. (C) Kaplan Meier survival analysis with log-rank test was performed; CAR-T vs HBSS *P* = .0018; CD4^+^ CAR-T vs HBSS *P* = .0018; CD4^+^ CAR-T ^+^ CAR-NK vs HBSS *P* = .016; CAR-NK vs HBSS *P* = .16. Data shown here is from one experiment. (D) Long-term survivor mice (from Figure 2C; CAR-T treated *n* = 5, CD4 + CAR-T *n* = 5, CD4 + CAR-T + CAR-NK *n* = 3, CAR-NK *n* = 1) were rechallenged with subcutaneous injection of 4e5 SB28EGFRvIII cells in the right flank. Treatment naïve, age matched C57BL6/J mice (*n* = 5) received identical subcutaneous injections as controls. Tumor burden was assessed by serial bioluminescence imaging and Kaplan Meier survival analysis was performed using Log-rank test (*P* = .0021).

In vitro, while both CAR-NK and wild-type (WT) NK-cells demonstrated effective cytotoxic activities against SB28 glioma cells that did not express EGFRvIII, the cytotoxic activity of CAR-NK cells was enhanced (*t* = 58 h, *P* < .001) when tumor cells expressed the CAR antigen EGFRvIII (Figure 2A).

We next tested in vivo therapeutic efficacy of CAR-NK cells in glioma-bearing mice.

Previous studies have demonstrated that CD4^+^ T cells could provide immunological help to NK-cells in the setting of infections.^24,25^ Therefore, we included a combination group of simultaneously administered CD4^+^ CAR-T cells and CAR-NK cells to test our hypothesis that CD4^+^ CAR-T cell-derived cytokines would support CAR-NK cell activation and persistence. In vitro, CD4^+^ CAR-T cells secreted IFNγ and GM-CSF upon co-culture with SB28EGFRvIII cells but low IL-2 and TNF-α production levels which did not increase significantly in the presence of SB28EGFRvIII cells (**Supplementary Figure 3A**). C57BL/6 mice bearing intracerebral SB28EGFRvIII glioma received a single intravenous (IV) dose of cyclophosphamide and fludarabine as the lymphodepleting regimen on day 10. Mice were then stratified to receive a single intravenous dose of (1) CAR-T cells (at 1:1 ratio of CD8^+^ and CD4^+^ CAR T cells), (2) CD4^+^ CAR-T cells, (3) CAR-NK-cells, and a combination of CD4^+^ CAR-T and CAR-NK-cells. While all mice treated with vehicle-only control (HBSS) showed progressive tumors and died by Day 41, mice receiving CAR-T cells (5 out of 6 mice) or CD4^+^ CAR-T (5 out of 6 mice) exhibited tumor clearance (**Figure 2B, C).** Despite similar tumor regression kinetics and survival benefits, the persistence of CAR-T cells was superior to CD4^+^ CAR-T cells in the peripheral blood on day 44 following adoptive transfer (**Supplementary Figure 3B****, left panel)**. The treatment with CAR-NK-cells alone did not result in a reduction in tumor burden in the majority of the mice (4 out of 5 mice) (**Figure 2B, C**). The co-administration of CD4^+^ CAR-T and CAR-NK cells resulted in tumor regression and improved survival in 3 out of 5 mice (**Figure 2B, C**). In the combination group (CD4^+^ CAR-T + CAR-NK), the efficacy is likely attributable to the CD4^+^ CAR-T cells, as the mice receiving CD4^+^ CAR-T cells alone showed a similar outcome. While CD4^+^ CAR-T cells produced IFN-γ and GM-CSF (**Supplementary Figure 3A**), these may not have supported the persistence and long-term function of CAR-NK-cells.

We then rechallenged the long-term survivor mice from all the cohorts with subcutaneous inoculation of SB28EGFRvIII cells to assess if they mounted anti-SB28EGFRvIII tumor response. Four of 5 mice that were initially treated with CAR-T cells showed slower tumor growth (**Figure 2D**) and CAR-T cells were detectable in the peripheral blood (**Supplementary Figure 3B****, right),** suggesting that the persistent CAR-T cells were functional. In contrast, CD4^+^ CAR-T-treated long-term survivor mice failed to reject subsequent flank tumors (**Figure 2D).** This observation is likely due to the low numbers of CD4^+^ CAR-T cells seen in the peripheral circulation (**Supplementary Figure 3B****, left).**

As locoregional delivery of CAR immune cells circumvents the limitation of cell trafficking, we next tested whether intra-tumoral injection of CAR-NK cells in mice with intracerebral SB28EGFRvIII mediates tumor regression. We chose the injection dose of 1 × 10^6^ CAR-immune cells to avoid cytokine-induced toxicity. While CAR-T cells demonstrated a trend toward improved survival (*P* = .035 and .066 against CAR-NK cells or PBS, respectively; **Supplementary Figure 3C**) and tumor regression in 2 out of 6 mice, CAR-NK cells failed to show improved survival.

### CAR-NKT-cells Mediate EGFRvIII-specific Direct Cytotoxic Effects and Secrete High Levels of IL-4, IL-17, and GM-CSF

**Figure 3.**
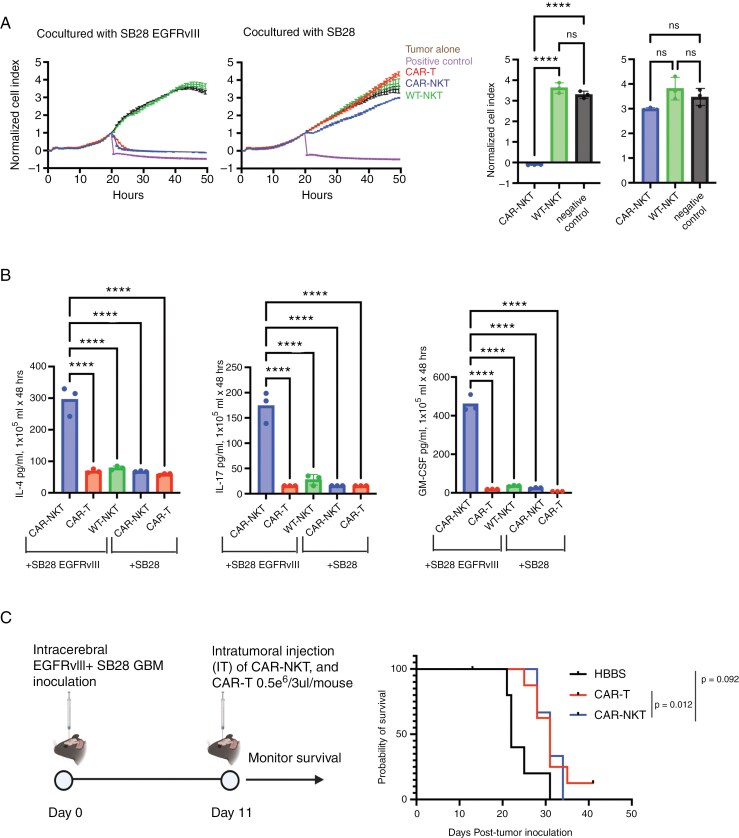
Characterization of CAR-NKT-cells. (A) Cytolytic activity of ex vivo expanded CAR-NKT evaluated using xCELLigence assay. The bar graphs on the right compare the anti-tumor effects of each immune cell at the 50-h time point (*n* = 3). (B) Production of cytokines (IL-4, IL-17, and GM-CSF) by CAR-NKT-cells when co-cultured with SB28EGFRvIII glioma cells (*n* = 3). (C) Survival after intratumoral (IT) administration of CAR-T or NKT-cells: HBSS IT group (MS = 22 days, *n* = 5), CAR-T cells IT (MS = 31 days, *n* = 10), and CAR-NKT IT (MS = 31 days, *n* = 3). (error bars show the mean with SD. ****P < .0001 by one-way ANOVA analysis followed by Tukey’s multiple comparison test).

NKT-cells have diverse functions that bridge between innate and adaptive immunity^26,27^ and CAR-NKT-cells are actively being evaluated in patients.^10,28^ As depicted in **Supplementary Figure 2A**, we isolated EGFRvIII-targeted CAR-NKT cells from the livers of Vav-Cre CAR-Tg mice and expanded them with anti-CD3/CD28 antibodies. In vitro, CAR-NKT-cells demonstrated EGFRvIII-specific cytotoxicity against SB28EGFRvIII cells, comparable to CAR-T-cells (**Figure 3A**, **Supplementary Figure 4A**). Notably, unlike CAR-NK and WT NK-cells, neither CAR-NKT nor WT NKT-cells showed more than a background level cytotoxic activity against parental SB28 cells (Figure 3A).

One major challenge of the current system is that we cannot harvest more than 0.1 million NKT-cells from a single mouse. During the 3-week culture course with repeated stimulations, the cell number gradually increased but did not exceed 0.4 million cells from a single mouse (**Supplementary Figure 4A, B**). Moreover, when we compared the cytotoxic activity levels of CAR-NKT cells at 1, 2, and 3 weeks of culture, the cells cultured for 1 week demonstrated the most potent anti-tumor effect (Figure 3A**and****Supplementary Figure 4C**). Hence, we decided to use the 1-week culture method moving forward.

NKT cells are known for their capacity to secret various cytokines. When we analyzed the cytokine levels in the supernatants obtained from the co-culture of SB28 glioma and NKT cells in the presence or absence of the CAR-mediated signaling, CAR-NKT cells co-cultured with SB28EGFRvIII cells showed a CAR-EGFRvIII-specific, significant increase in the secretion of IL-4, IL-17, and GM-CSF (Figure 3B).

However, our in vivo experiments with mice bearing intracerebral SB28EGFRvIII gliomas faced a challenge in preparing sufficient numbers of NKT cells. We obtained less than two million cells from fifteen donor Vav-Cre CAR-Tg mice following the optimized culture and expansion. Therefore, we had only three mice receiving a single intratumoral infusion of CAR-NKT cells (5 × 10^5^ cells/mouse), while we had 10 and 5 mice/group for CAR-T and control PBS treatments, respectively (Figure 3C). CAR-T- and CAR-NKT-treated mice showed the same median survival time (31 days), and the CAR-T-treated mice showed a statistically significant prolongation of survival compared with the HBSS-treated group (median survival time: 22days, *P* = .012) (Figure 3C).

### Combination of CAR-T and CAR-NKT-cell Shows Enhanced Anti-tumor Efficacy in the SB28EGFRvIII Mouse Model

**Figure 4.**
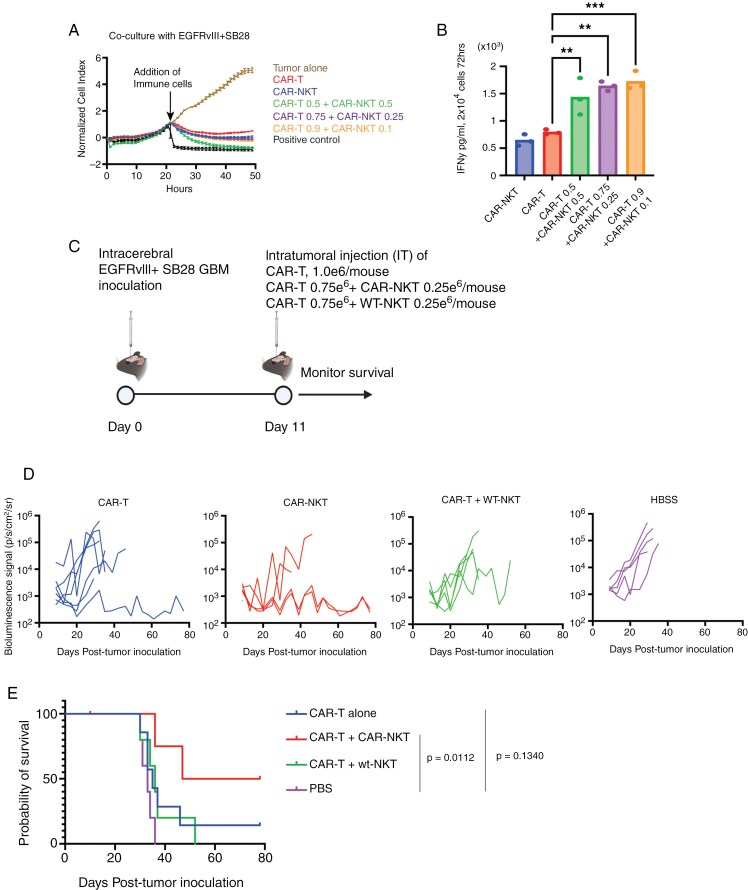
Enhanced anti-tumor effects of combined CAR-NKT and CAR-T-cell therapy. (A) Cytolytic activity of ex vivo expanded CAR-NKT-cells evaluated in combination with CAR T-cells using xCELLigence assay (*n* = 3). Ratio of target to effector cells was 1:4. In these experiments shown in Figure 4, CD3-CAR-T cells (a mixture of CD4⁺ and CD8⁺ cells) are referred to as CAR-T. (B) Production of IFNγ by CAR-NKT cells alone or in combination with CAR T cells when co-cultured with SB28-EGFRvIII tumor cells (*n* = 3, Error bars show the mean with SD. **P < .01; ***P < .001 by one-way ANOVA analysis followed by Tukey’s multiple comparison test). (C) Schematic of the treatment protocol for the survival study using male mice. (D) Tumor size was measured by longitudinal luciferase BLI as the number of photons per second per square centimeter per steradian (p/s/cm^2^/sr). (E) Kaplan–Meier curves: CAR-T group (MS = 35 days, *n* = 7), CAR-T ^+^ CAR-NKT (MS = 50 days, *n* = 4), CAR-T ^+^ wt-NKT (MS = 36 days, *n* = 5), and HBSS (MS = 33 days, *n* = 5). *P* = .0112 between the CAR-T ^+^ CAR-NKT group and HBSS group.

The high level of cytokine production by CAR-NKT-cells (Figure 3B) led us to hypothesize that CAR-NKT-derived cytokines would enhance the effectiveness of CAR-T cells when CAR-NKT and CAR-T cells are co-delivered in vivo. When tested in vitro, the mixture of CAR-T and CAR-NKT cells in various ratios did not show enhanced levels of cytotoxicity against SB28EGFRvIII cells compared to treatments with CAR-T or CAR-NKT alone (Figure 4A, **Supplementary Figure 5A**). On the other hand, analysis of IFNγ levels in the culture medium revealed significantly higher IFNγ levels in the combination groups than in either cell type alone (with the constant total cell numbers across the groups, Figure 4B). However, at E: T ratio = 1:1, IFNγ production levels in the 9:1 CAR-T:CAR-NKT ratio were lower than in the 3:1 ratio (**Supplementary Figure 5B**). Based on these findings and considering the availability of NKT-cells, we decided to proceed with a survival study by administering 0.75 million CAR-T- and 0.25 million CAR-NKT cells per mouse.

Mice bearing intracerebral SB28EGFRvIII tumors were stratified to receive an intratumoral injection of either (1) 1 million CAR-T cells, (2) 0.75 million CAR-T and 0.25 million CAR-NKT cells, (3) 0.75 million CAR-T and 0.25 million WT-NKT cells, or (4) HBSS, and monitored the survival (Figure 4C). We deliberately used low dose of CAR-T cells (1e6 cells) so that the CAR-T therapy by itself underperforms and the potency of CAR-NKT cells in combination with CAR-T cells can be assessed. CAR-T cell therapy alone did not significantly extend the survival (Figure 4D–E), only one of 7 mice treated with CAR-T cells survived for at least 78 days (*P* = .1340). However, the combination of CAR-T- and CAR-NKT-cells significantly improved the survival of mice compared to the HBSS group (*P* = .011), with two of four mice surviving for at least 78 days (Figure 4D-E). These data suggest the potential of CAR-T and CAR-NKT combination therapy to enhance the efficacy of single-cell type CAR cell therapy, warranting further exploration of combinatory cellular immunotherapies.

### EGFRvIII-targetted CAR-mac Produce Elevated Levels of TNFα in an Antigen-specific Manner but Fail to Show Enhanced Phagocytotic or Antigen-presenting Functions

**Figure 5.**
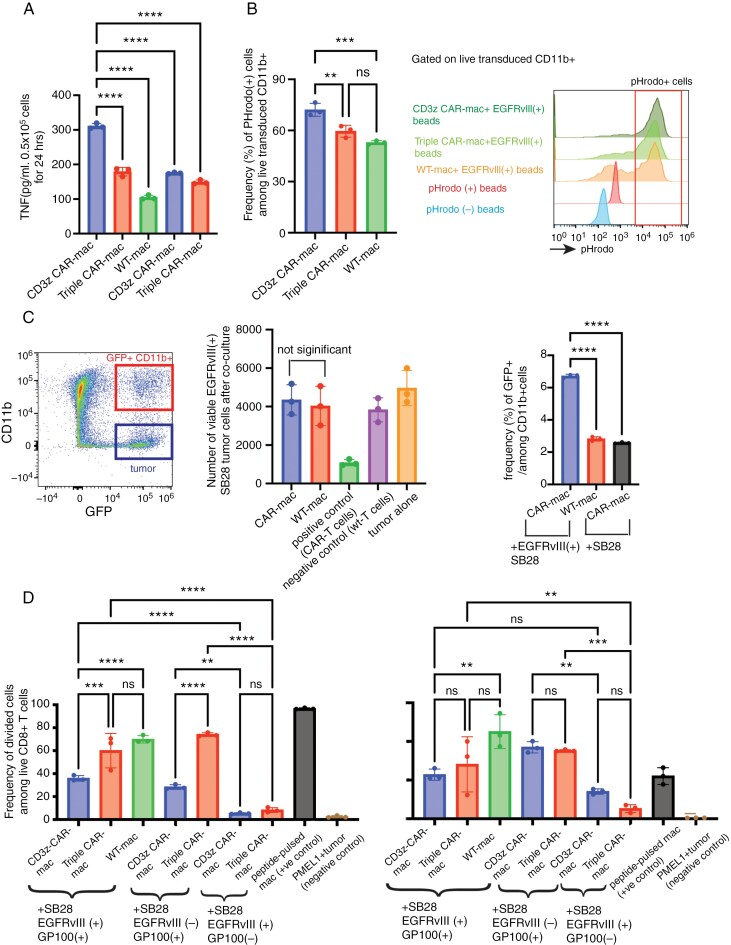
Phagocytotic function and cross-presentation by CAR-mac. (A) TNFα concentration by ELISA after co-culturing EGFRvIII-targeted CAR-mac with beads bound to EGFRvIII. (B) Comparison of phagocytosis of pHrodo-labeled EGFRvIII-bound beads by CAR-mac with different intracellular domains. Histograms show the gating strategy for pHrodo-positive cells. (C) Co-culture of EGFRvIII-targeted CD3z CAR-mac and GFP-expressing SB28-EGFRvIII or parental SB28 glioma cells (see Supplementary Figure 2C for experimental details). The FACS dot plot shows the gating strategy. GFP-positive and CD11b-positive cells were considered macrophages phagocytosed SB28 cells, and CD45-negative CD11b-positive cells were considered tumor cells. Bar graphs show a comparison of the number of surviving tumor cells and phagocytosis-positive cells across each group after co-culture. There are no significant differences in the number of surviving tumor cells between the CAR-mac and WT-mac groups, regardless of stimulation. (D) Cross-presentation of the glioma cell-derived GP100 epitope by CAR mac. CAR mac cells were first co-cultured with SB28 glioma cells with or without the GP100 expression. Then, those CAR mac were co-cultured with Pmel1-T-cells (see Supplementary Figure 2D for experimental details) to evaluate IFNγ production by ELISA and T cell proliferation by Cell Trace. (*n* = 3, Error bars show the mean with SD. **P < .01; ****P < .0001 by one-way ANOVA analysis followed by Tukey’s multiple comparison test).

A growing body of literature has documented the efficacy of CAR macrophages (CAR-mac) in targeting a diverse array of tumor types.^29–32^ Regarding the choice of the intracellular domain in the CAR construct, the majority of investigators have successfully utilized the CD3z^30^ MegF10, FcRγ^31^ or CD147 domains. In our work, leveraging the CD3z-CD28-4-1BB tri-domain architecture in the Vav-Cre CAR-Tg mouse model, we have developed syngeneic CAR-mac endowed with CD3z-CD28-4-1BB by culturing Vav-Cre CAR-Tg mouse bone marrow-derived CD11b^+^ cells with GM-CSF (**Supplementary Figure 2B**). Our initial investigations sought to delineate the distinctions in functionality between CAR-mac endowed solely with the CD3z domain (referred to as CD3z CAR-mac) and those that express the CD3z-CD28-4-1BB domain, herein termed Triple CAR-mac. We stimulated CAR-macs with EGFRvIII recombinant protein-bound beads (EGFRvIII-bound beads). EGFRvIII stimulation induced higher levels of TNFα production in CD3z CAR-mac than Triple CAR-mac (Figure 5A). The EGFRvIII-specific enhancement of TNFα production indicates the antigen-specific function of the CARs in these cells. Additionally, phagocytic activity, assessed using pHrodo-labeled EGFRvIII-bound beads, was higher in CD3z CAR-mac relative to Triple CAR-mac and control WT-mac (Figure 5B). When we conducted another experiment by co-culturing CAR-mac and green fluorescence protein (GFP)-expressing SB28 cells (with or without EGFRvIII expression), CAR-mac did not show potent anti-SB28 effects (evaluated by the number of viable tumor cells). However, a higher percentage of GFP^+^CD11b^+^ cells (ie, myeloid cells that engulfed GFP^+^ SB28 cells) was observed in the CAR-mac when co-cultured with SB28EGFRvIII gliomas, suggesting antigen-specific phagocytosis, compared to control groups (**Figure 5C**).

A previous study has demonstrated that CAR-mac can present tumor antigens as antigen-presenting cells after phagocytosing tumor cells.^30^ Using our syngeneic model, we assessed whether the EGFRvIII-CAR signaling could enhance the cross-presentation of tumor antigens using SB28EGFRvIII-gp100 tumor cells expressing the H-2Db-restricted pmel epitope derived from gp100.^33,34^ The pmel epitope allows sensitive and reliable evaluation of antigen-specific T-cell response using pmel-1 T-cells.^35,36^ We initially co-cultured SB28EGFRvIII-gp100 cells with CAR-mac, allowing the antigen uptake, then subsequently those CAR-mac and CellTrace-labeled PMEL-1 T-cells to evaluate the antigen-driven proliferation of PMEL-1 T-cells and IFNγ production (**Figure 5D**, **Supplementary Figure 2C**). Compared to the control SB28EGFRvIII cells without gp100, SB28EGFRvIII-gp100 cells induced significantly higher levels of T-cell division and IFN-γ production in all macrophage groups tested, indicating that the macrophages effectively presented the pmel epitope derived from SB28EGFRvIII cells and induced specific responses in pmel-1 T-cells. However, among the conditions with SB28EGFRvIII-gp100 cells, the EGFRvIII-CAR signal did not induce a higher level of T-cell response compared to control groups, without any observable enhancement attributable to the CAR constructs (**Figure 5D**). These observations imply that while CAR-mac is expected to phagocytose tumor cells in an antigen-specific manner (**Figure 5C**), the EGFRvIII-CAR signal does not enhance macrophages’ capacity to effectively cross-present the antigen.

### Ineffectiveness of EGFRvIII-targeted CAR-mac In Vivo Despite Transient Impacts

**Figure 6.**
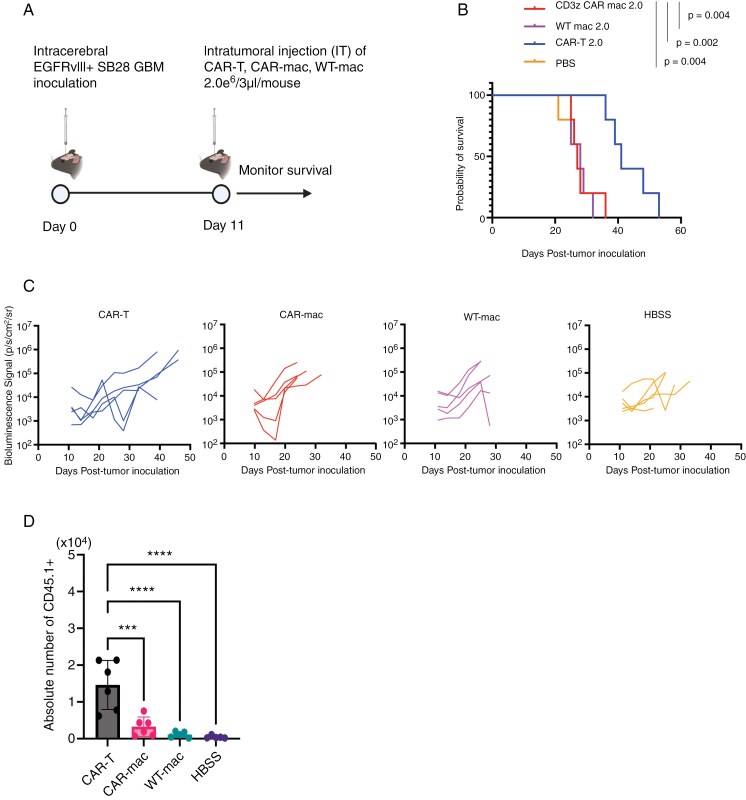
CAR-mac fails to extend survival in brain tumor mice and does not sustain presence within brain tumors. (A) Schematic of the treatment protocol for the survival study with the SB28-EGFRvIII glioma cells. (B) Survival of mice bearing intracerebral SB28-EGFRvIII gliomas treated with IT injection of 2 × 10^6^ CAR-mac. Kaplan-Meier curves: CAR-T group (MS = 41 days, *n* = 5), CAR-mac (MS = 27 days, *n* = 5), wt-mac (MS = 28 days, *n* = 5), and HBSS (MS = 27 days, *n* = 5). (C) Tumor size was measured by longitudinal luciferase BLI as the number of photons per second per square centimeter per steradian (p/s/cm2/sr). (D) The number of injected (CD45.1^+^) macrophages in brain tumors 4 days after administration of CAR-mac to an EGFRvIII-positive intracranial tumor mouse model. (*n* = 6, Error bars show the mean with SD. ***P < .001; ****P < .0001 by one-way ANOVA analysis followed by Tukey’s multiple comparison test).

Despite the modest functions of EGFRvIII-targeted CAR-mac in vitro, the significant infiltration of myeloid cells within glioma suggests a potential for in vivo efficacy.^37,38^ We administered 2 million CAR-mac intratumorally into a mouse brain tumor model and monitored survival outcomes (**Figure 6A**). Since the CCL2/CCR2 axis plays a role in macrophage recruitment to tumors, we evaluated multiple culture conditions and confirmed that the same GM-CSF-based culture ensures the highest CCR2 levels, and hence, we used the same culture conditions for in vivo experiments^39,40^ (**Supplementary Figure 6A, B**). Although CAR-T-cells significantly extended survival, CAR-mac did not exhibit any therapeutic effect (**Figure 6B**). Notably, a temporary BLI signal reduction in two of five mice (between Day 10–20) suggested a potential transient effect of CAR-mac early after administration (**Figure 6C**). Although we evaluated various administration routes, including IV and intracerebral injections, we observed suboptimal persistence of CAR-mac within brain tumors (**Figure 6D and****Supplementary Figure 7**).

## Discussion

Our current study utilizing the Vav-Cre CAR-Transgenic mouse model allowed us to investigate the potential of NK-, NKT-, and macrophage-based CAR therapies in the SB28 syngeneic mouse glioma model, thereby providing significant insights into the development of CAR-engineered immune cells.

Our findings demonstrate that CAR-NKT cells, similar to CAR-T cells, can exert potent anti-tumor effects through direct CAR-mediated signaling. Furthermore, CAR-NKT cells secrete a diverse array of cytokines in an antigen-specific manner. Our study also suggested that combination therapy with CAR-NKT and CAR-T cells might offer superior anti-tumor activity compared to treatment with CAR-T cells alone. Despite the small number of mice treated with CAR-NKT cells due to the limited cell availability, our data suggest that CAR-NKT cells could play a crucial role in expanding the therapeutic arsenal of CAR-based treatments.

CAR-NK cells have distinct advantages over CAR-T cells in three ways. First, recent clinical trials with allogeneic CAR-NK cells have shown an excellent safety profile in terms of low frequency of graft-vs-host disease (GVHD), cytokine release syndrome (CRS), and neurotoxicity.^41^ Second, CAR-NK cells can also eliminate tumor cells in an antigen-independent manner and thus theoretically circumvent the need for the target antigen to be homogenously expressed on the tumor. Third, starting source material for manufacturing CAR-NK cells can range from PBMCs, umbilical cord blood, iPSCs, and the NK92 cell line, and all these options have the advantage of being “off-the-shelf” therapeutic.^42^

On the other hand, compared to CAR-T cells, there are two significant issues with CAR-NK-cells: (1) short in vivo persistence and (2) suboptimal ex vivo expansion of NK cells to obtain a large number for clinical studies. Strategies to improve the lifespan of CAR-NK cells include expression of membrane-bound or secreted IL-15 alongside CAR construct, or co-administration of an IL-15 superagonist to allow the post-infusion expansion of CAR-NK-cells in vivo.^43–45^ The constitutive expression of IL-15 combined with genetic deletion of a negative regulator of cytokine (CISH) in CAR-NK cells has proven to be efficacious in the pre-clinical lymphoma model.^46^ Another approach to improve in vivo persistence is to program memory-like NK-cells using a cytokine cocktail of IL-12 and IL-18^21^ as we did in our current study using spleen-derived transgenic CAR-NK cells. However, intratumoral (IT) or IV-delivered CAR-NK cells did not mediate effective anti-tumor response in an orthotopic GBM model in immunocompetent mice. Therefore, we may need to provide IL-2, IL-15, or IL-15 superagonist when we treat immunocompetent mice with CAR-NK cells.

Our CAR Tg mouse system allows us to evaluate combinatory approaches of multiple CAR-expressing immune cells. A previous study utilizing cord blood-derived CAR-NK and CAR-T cells in the setting of multiple myeloma and non-Hodgkin lymphoma reported the added benefits of CAR-NK cells in attenuating CAR-T cell exhaustion and promoting early activation of CAR-T cells.^47^ We hypothesized that CD4^+^ CAR-T cells would provide cytokines, such as IL-2 and IL-21 for improving CAR-NK cell survival and persistence. However in our study, CD4^+^ CAR-T cells did not secrete enhanced levels of IL-2 (**Supplementary Figure 3A**) upon exposure to glioma cells and failed to improve the therapeutic efficacy of CAR-NK cells. Although CD4^+^ CAR-T cells secreted IFNγ and GM-CSF, these cytokines may not have impacted the CAR-NK cell persistence.

Our exploration of EGFRvIII-targeted CAR-mac in the SB28 glioma model presents a more complex scenario. Although previous studies using CAR-mac in PDX models showed promising results,^30^ our study with the syngeneic mouse glioma model did not reveal a significant anti-tumor effect of CAR-mac. Genetic differences between humans and mice result in significant differences in macrophage function and response.^48^ Zhang et al. demonstrated that HER2-targeted CAR expressed by Raw264.7 cell line-derived murine macrophages showed limited efficacy in vitro against HER2-overexpressing 4T1 cells but exhibited anti-tumor effects in vivo.^49^ A recent study using bone marrow-derived macrophages engineered with a CAR incorporating the intracellular toll/IL-1 receptor (TIR) domain targeting GPC3-overexpressing Hepa1-6 cells showed effectiveness in a syngeneic mouse model.^50^ In our study, the use of the CD3z intracellular domain resulted in elevated TNFα production levels in the EGFRvIII-CAR signaling-specific manner. The CD3z intracellular domain in CAR-mac has been shown to be effective in multiple studies.^30,50^ Our evaluations of phagocytic and antigen-presentation activities showed that the macrophages we used had high levels of those activities, which were not enhanced by the CAR expression and EGFRvIII. In our hands, CAR-mac did not persist well in vivo, suggesting a need to improve the persistence, especially in the glioma environment.

### Limitations of the Study

While our novel transgenic CAR mouse model provides an effective tool to evaluate multiple CAR-expressing immune populations and their combinations, more comprehensive evaluations remain to be performed with multiple syngeneic glioblastoma models. While we selected EGFRvIII as the CAR target, future studies should also evaluate CARs targeting other antigens, including tumor-associated antigens endogenously expressed in murine glioma models. Furthermore, while the CD3z-CD28-4-1BB (“triple”) intracellular signaling domain of our EGFRvIII CAR, which was built in our transgenic mouse system, is optimal for T cells, our future studies should evaluate other intracellular co-stimulatory domains for CAR-expressing macrophage and NK cells. The technical challenge of obtaining large numbers of CAR-NKT cells precluded us from performing dose optimization studies, testing multiple dosing regimens and evaluating CAR infiltration in the tumor microenvironment.

In summary, as the first study evaluating multiple immune cell types expressing CAR in a syngeneic mouse model, our data provide valuable insights into the field, highlighting the promising potential of CAR-NKT cells in combination therapies and the challenges of CAR-macrophage application in brain tumors. These findings also underscore the need for further exploration and optimization of CAR-based therapies to enhance their efficacy and expand their applicability in the treatment of cancer.

## Supplementary Material

Supplementary material is available online at *Neuro-Oncology Advances* (https://academic.oup.com/noa).

vdaf074_suppl_Supplementary_Figures_S1-S7

## Data Availability

The data will be made available upon reasonable request.
