# Supplementary material for: Comparative evaluation of CAR-expressing T-, NK-, NKT-cells, and macrophages in an immunocompetent mouse glioma model
Source: Neurooncol Adv. 2025 Apr 12;7(1):vdaf074. doi: 10.1093/noajnl/vdaf074 (PMC12080550; doi:10.1093/noajnl/vdaf074)
Supplement: vdaf074_suppl_Supplementary_Figures_S1-S7 [file vdaf074_suppl_supplementary_figures_s1-s7.zip › vdaf074_suppl_Supplementary_Figures_1-7/Supplementary Figure Legends.docx]

**Supplementary Figure Legends**

**Supplementary Figure 1: Gating strategy for the CAR expression in immune cell populations and pre-infusion composition of CAR+ immune populations**. A) Illustrating the flow cytometry gating strategy employed in Figure 1B. B) Pre-infusion purity of CAR-T, CAR-NK, CAR-NKT, CAR CD4+ T -cells, and CAR-macrophages.

**Supplementary Figure 2: Ex-vivo expansion schema for immune cell populations – CAR-NKT, CAR-T CAR-NK, and CAR-macrophages**. A, B) Illustrating the *in vitro* culture method for CAR-NKT, CAR-T CAR-NK, and CAR-macrophages. C, D) Schema for the experimental setup of CAR-macrophage phagocytosis and cross-presentation assay.

**Supplementary Figure 3: Assessment of cytokines produced by CD4^+^ CAR-T cells and anti-tumor effect of CAR-NK cells following intra-tumoral administration.** A) The supernatant of SB28EGFRvIII- CD4^+^ CAR-T cell co-culture was analyzed for IL-2, IFNγ, TNFα, and GM-CSF by Luminex. Pairwise comparison was done using a one-way ANOVA test and indicated on the graph. B) Left panel: C57BL6/J mice bearing intracerebral SB28EGFRvIII glioma received single intravenous dose of either CAR-T (3x10^6^ cells per mouse) or CD4^+^ CAR-T (3x10^6^ cells per mouse) following lymphodepletion. The presence of CD3 CAR-T, and CD4^+^ CAR- T cells in the peripheral blood on day 44, assessed by flow cytometry. The peripheral blood was drawn from two mice. For CAR-T treated cohort, live cells were gated on CD45.1 and CD3. For CD4^+^ CAR-T treated cohort, live cells were gated on CD45.1, CD3, and CD4 positive cells. Paired two-tailed Student’s *t*-test p= 0.0167 CAR-T vs CD4^+^CAR-T. Right panel: CAR-T treated long-term survivor mice from Figure 2C were rechallenged with subcutaneous SB28EGFRvIII tumor. Peripheral blood from 4 mice was assessed for the presence of CAR-T cells, live cells were gated on CD45.1 and CD3.

C.) C57BL6/J mice bearing intracerebral SB28EGFRvIII glioma received single intra-tumoral dose of either CAR-T (1x10^6^ cells per mouse, n=6) or CAR-NK-cells (1x10^6^ per mouse, n=7). Control mice (n=5) received intra-tumoral injection of HBSS. Tumor size was monitored by serial bioluminescence imaging and survival was assessed by Kaplan Meir curve (Log-rank test: CAR-T vs CAR-NK; p =0.035, CAR-T vs HBSS; p=0.066). Data is shown from one experiment.

**Supplementary Figure 4: Assessment of anti-tumor effect and cell dynamics of CAR-NKT-cells in cultivation over time.** A) Schematic of the experimental setup. B) Cell counts observed throughout the cultivation period. C) Bar graph showing the comparison of cytotoxic activity levels of CAR-NK-T cells against SB28EGFRVIII glioma at one, two, and three weeks of culture. (Error bars show the mean with SD. **P < 0.01; ***P <0.001; ****P <0.0001 by one-way ANOVA analysis followed by Tukey’s multiple comparison test).

**Supplementary Figure 5: Assessment of anti-tumor effect and IFNγ secretion of the combination therapy at a 1:1 E:T ratio**. A) Evaluation of the anti-tumor effectiveness of combination therapy at an E:T ratio of 1:1 using xCELLigence. B) IFNγ levels in culture supernatants derived from the experiment in part A by ELISA.

**Supplementary Figure 6: Changes in the frequency of CCR2-positive cells across the different culture methods.** A, B) The line graph illustrates the percentage of CCR2-positive cells in CAR-mac (upper panel) and WT-mac (lower panel) during the course of each culture method.

**Supplementary Figure 7: Distribution of CAR-mac post-ICV and IV administration in intracerebral SB28EGFRvIII gliomas**. A, B) Number of CAR-mac in the brain at day 4 post IV or ICV administration of 1.8 × 10^6^ cells. The numbers of IV-administered CAR-T cells (1.8 × 10^6^ cells were infused) are shown as positive control.
