# Supplementary figures and images for: Comparative evaluation of CAR-expressing T-, NK-, NKT-cells, and macrophages in an immunocompetent mouse glioma model
Source: Neurooncol Adv. 2025 Apr 12;7(1):vdaf074. doi: 10.1093/noajnl/vdaf074 (PMC12080550; doi:10.1093/noajnl/vdaf074)

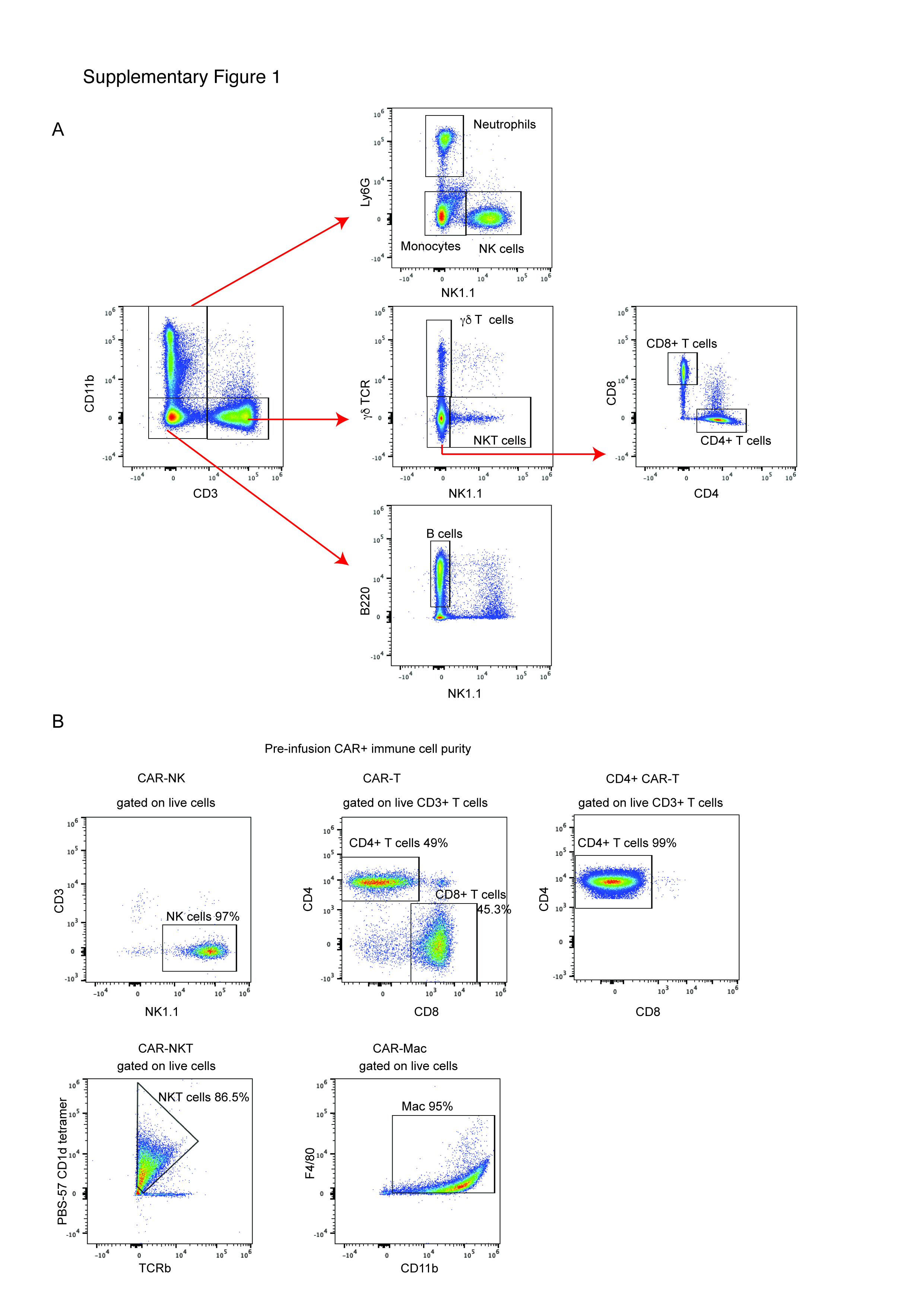

Supplement: vdaf074_suppl_Supplementary_Figures_S1-S7 [file vdaf074_suppl_supplementary_figures_s1-s7.zip › vdaf074_suppl_Supplementary_Figures_1-7/Supplementary fig 1.tif]

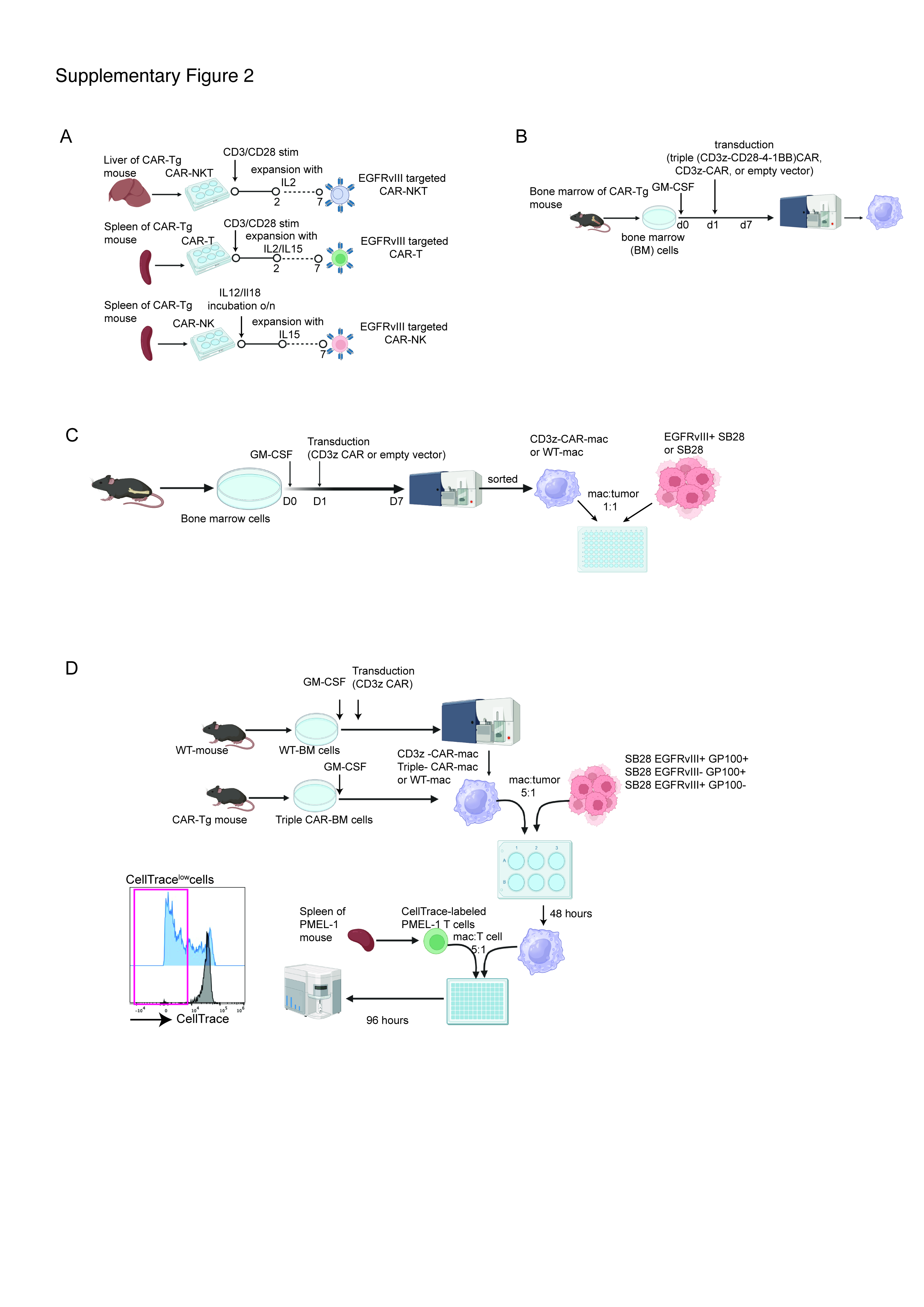

Supplement: vdaf074_suppl_Supplementary_Figures_S1-S7 [file vdaf074_suppl_supplementary_figures_s1-s7.zip › vdaf074_suppl_Supplementary_Figures_1-7/Supplementary Fig 2.tif]

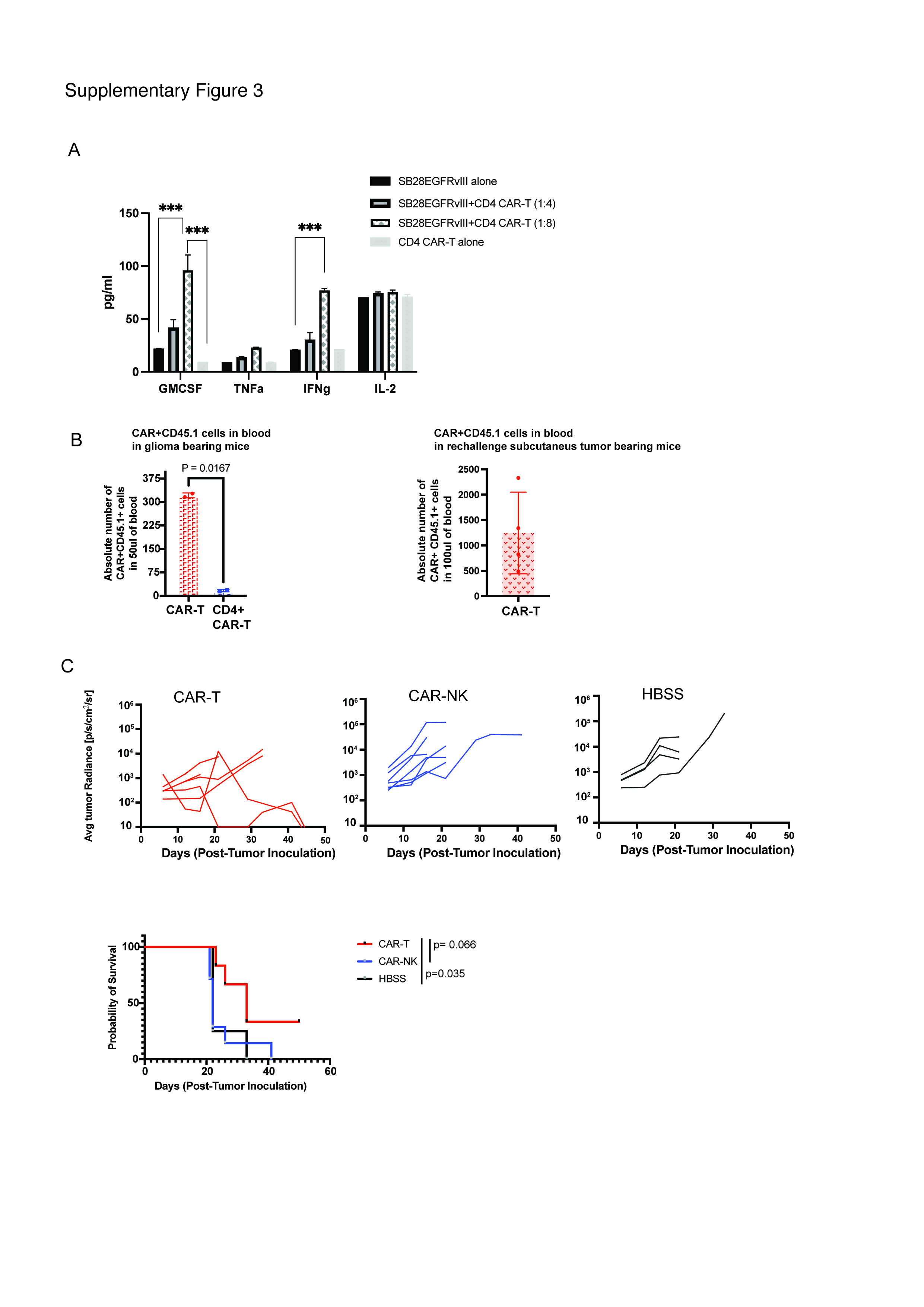

Supplement: vdaf074_suppl_Supplementary_Figures_S1-S7 [file vdaf074_suppl_supplementary_figures_s1-s7.zip › vdaf074_suppl_Supplementary_Figures_1-7/Supplementary fig 3.tif]

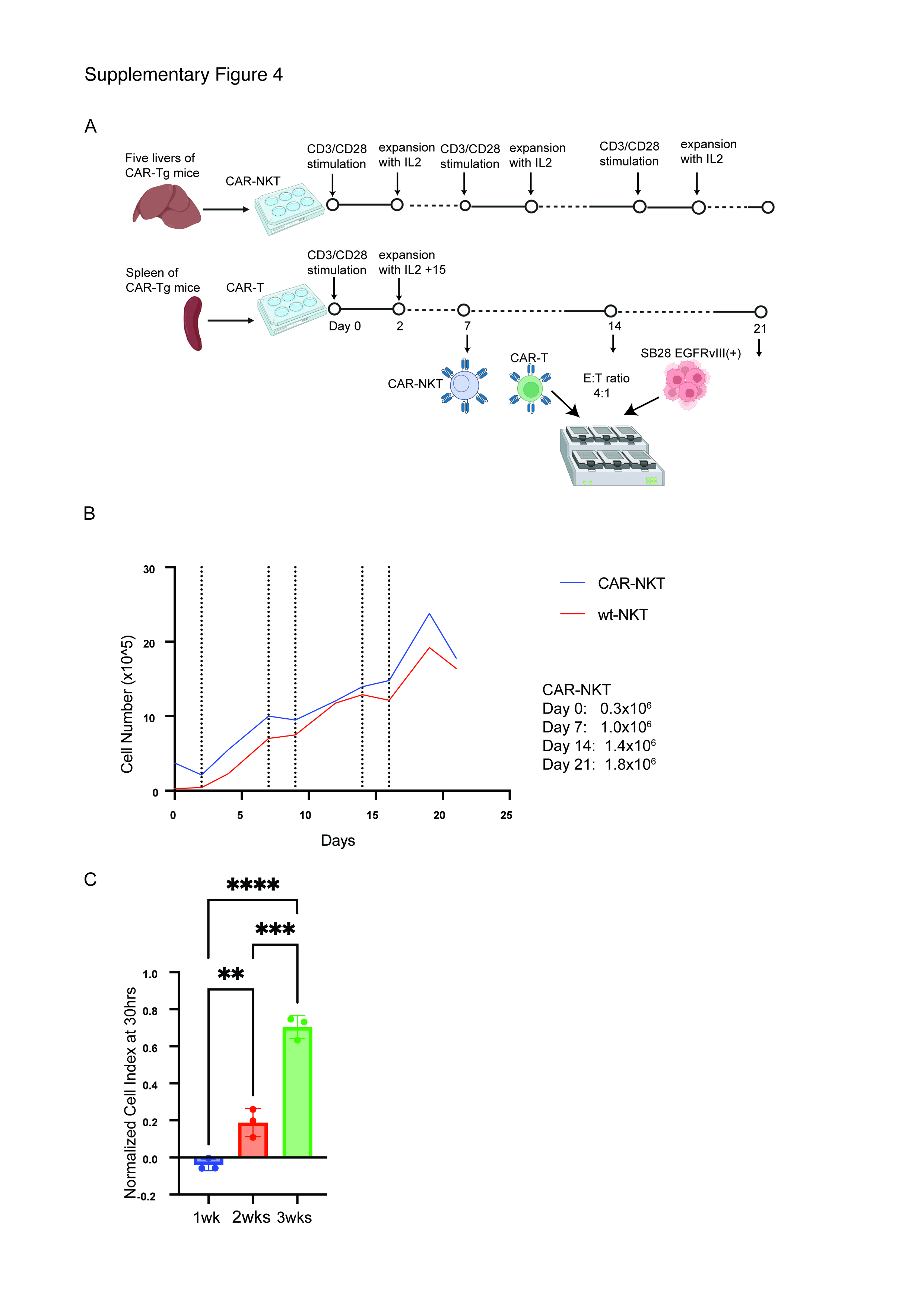

Supplement: vdaf074_suppl_Supplementary_Figures_S1-S7 [file vdaf074_suppl_supplementary_figures_s1-s7.zip › vdaf074_suppl_Supplementary_Figures_1-7/Supplementary Fig 4.tif]

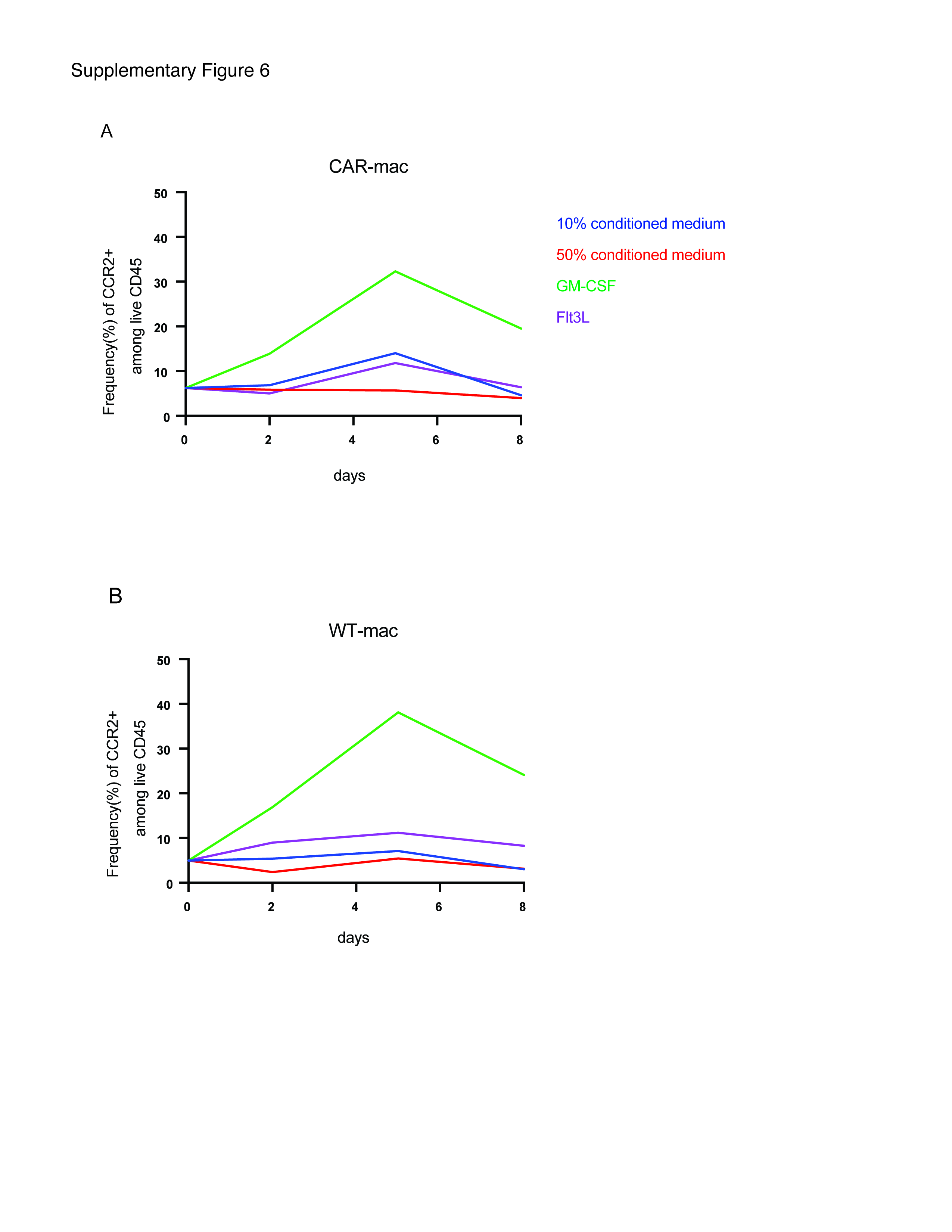

Supplement: vdaf074_suppl_Supplementary_Figures_S1-S7 [file vdaf074_suppl_supplementary_figures_s1-s7.zip › vdaf074_suppl_Supplementary_Figures_1-7/Supplementary Fig 6.tif]

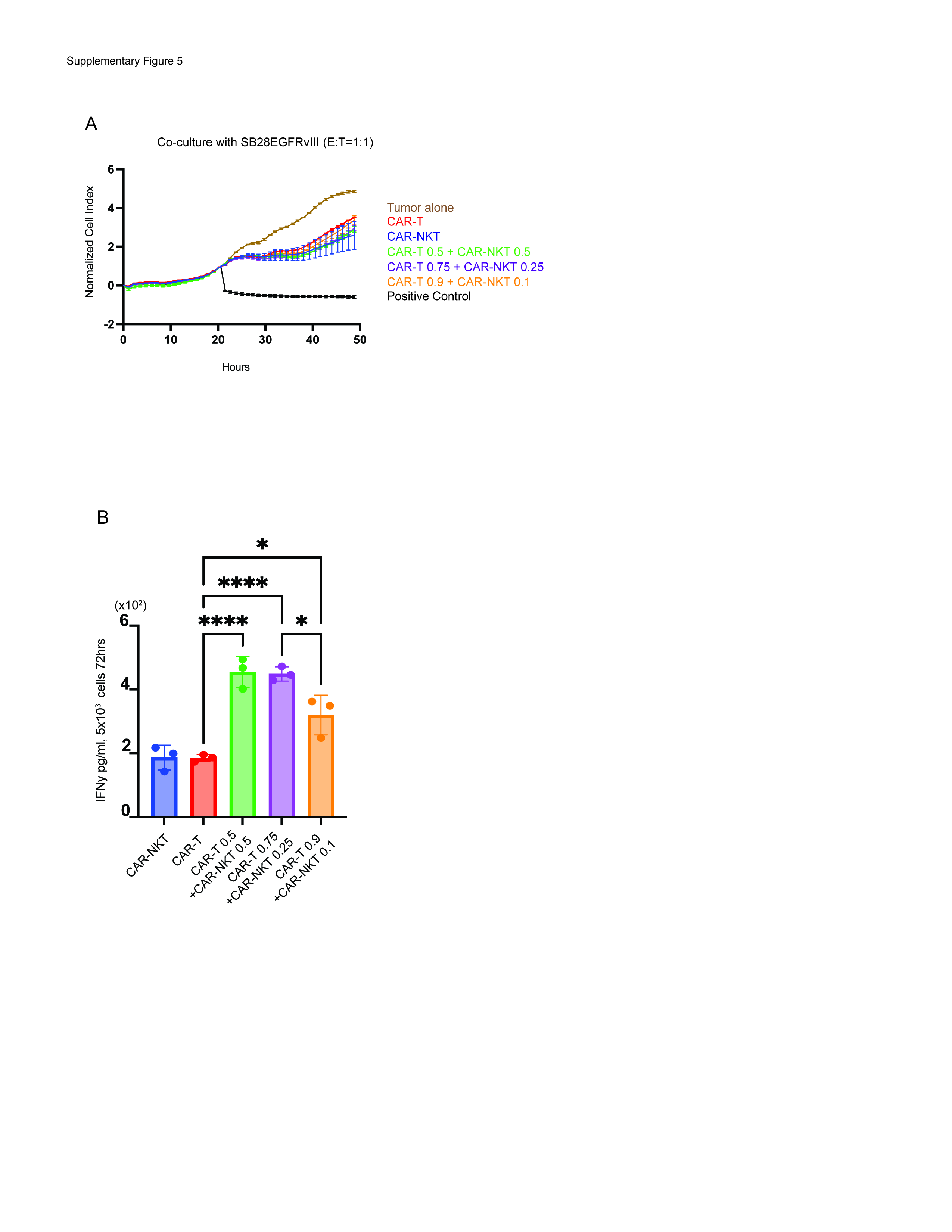

Supplement: vdaf074_suppl_Supplementary_Figures_S1-S7 [file vdaf074_suppl_supplementary_figures_s1-s7.zip › vdaf074_suppl_Supplementary_Figures_1-7/Supplementary Figure 5.tif]

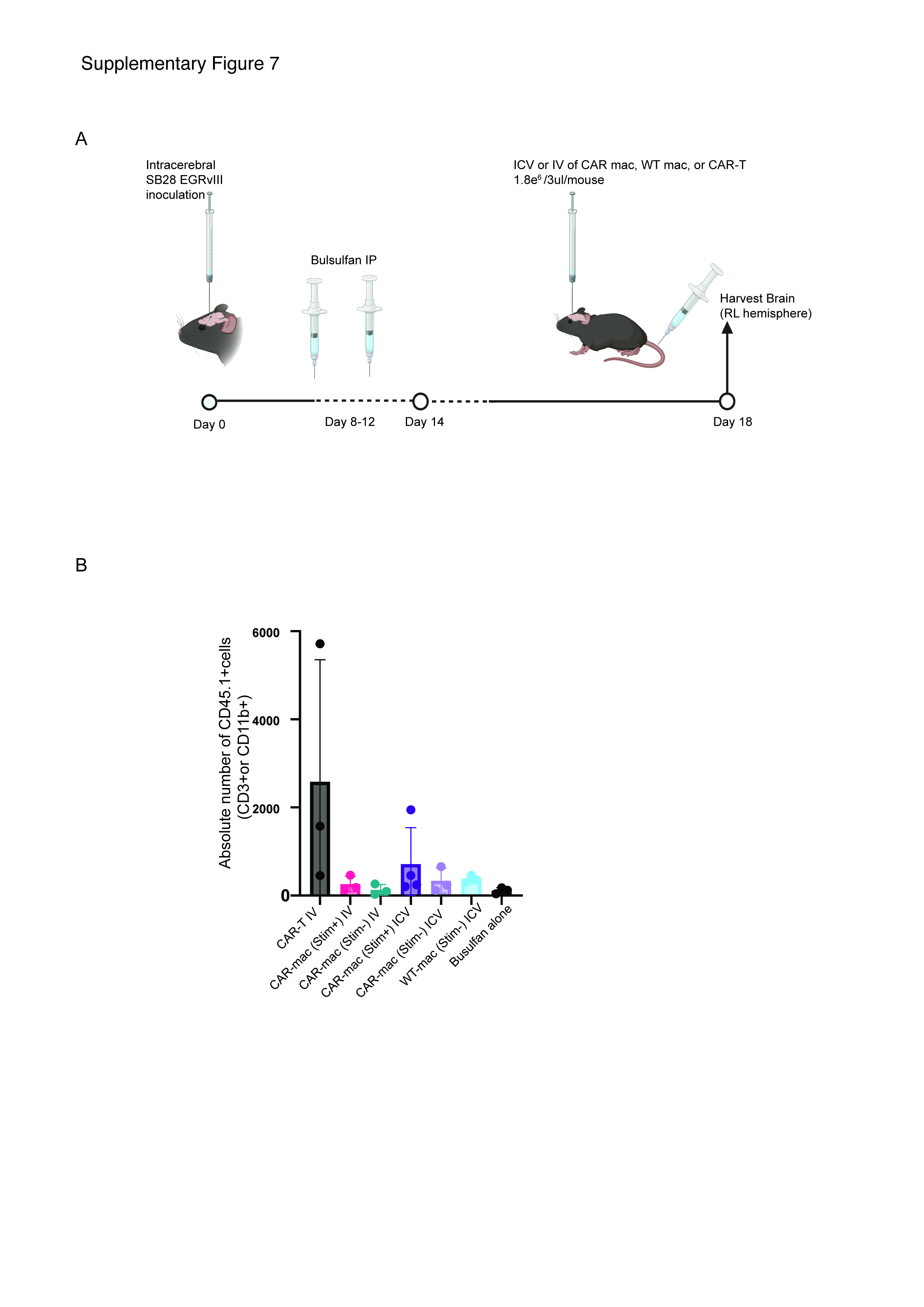

Supplement: vdaf074_suppl_Supplementary_Figures_S1-S7 [file vdaf074_suppl_supplementary_figures_s1-s7.zip › vdaf074_suppl_Supplementary_Figures_1-7/Supplementary Figure 7.tif]
